# Supplementary material for: DeNTNet: Deep Neural Transfer Network for the detection of periodontal bone loss using panoramic dental radiographs
Source: Sci Rep. 2019 Nov 26;9:17615. doi: 10.1038/s41598-019-53758-2 (PMC6879527; doi:10.1038/s41598-019-53758-2)
Supplement: Supplementary file 1 — Supplementary Information [file 41598_2019_53758_MOESM1_ESM.pdf]

DeNTNet : Deep Neural Transfer Network for the detection of periodontal bone loss using panoramic dental radiographs

Jaeyoung Kim[1], Hong-seok Lee[1], In-Seok Song[2,*], and Kyu-Hwan Jung[1,*]

[1]VUNO Inc., 6F, 507, Gangnam-daero, Seocho-gu, Seoul, South Korea
[2]Department of Oral and Maxillofacial Surgery, Korea University Anam Hospital, Seoul, South Korea
[*]densis@korea.ac.kr, khwan.jung@vuno.co

ABSTRACT

This document provides additional data and figures to supplement the paper "DeNTNet : Deep Neural Transfer Network for the detection of periodontal bone loss using panoramic dental radiographs ."

Data Statistics

Data Split		Training Set	Validation Set	Test Set
Number of Patients		11,189	190	800
Sex	Male	5,482(49%)	106(56%)	384(48%)
	Female	5,707(51%)	84(44%)	416(52%)
Age Group	20-29	1,903(17%)	34(18%)	96(12%)
	30-39	1,231(11%)	30(16%)	64(8%)
	40-49	1,343(12%)	35(18%)	120(15%)
	50-59	2,126(19%)	36(19%)	184(23%)
	60-69	2,394(21%)	17(9%)	176(22%)
	≥70	2,237(20%)	38(20%)	160(20%)
Patient-level Prevalence	Incisors	2,674(24%)	58(31%)	217(27%)
	Canines	2,747(25%)	57(30%)	201(25%)
	Premolars	3,285(34%)	72(38%)	284(36%)
	Molars	3,848(29%)	89(33%)	363(35%)
	Total	4,415(39%)	92(48%)	410(51%)
Tooth-level Prevalence	Incisors	13,065(10%)	273(12%)	939(10%)
	Canines	7,173(16%)	137(18%)	468(15%)
	Premolars	14,037(16%)	316(21%)	1,041(16%)
	Molars	14,333(11%)	353(15%)	1,327(14%)
	Total	48,608(13.5%)	1,079(18%)	3,775(15%)

Table 1. Statistics of training, validation, and test datasets. The datasets were obtained from various sex and age patient group. The patient-level and tooth-level prevalence is also described for all teeth types including incisors, canines, premolars, and molars.

Architecture Details of DeNTNet

ROI and lesion segmentation model

Layer	Output Shape	Connected to	Kernel size	Activation	Batch Normalization
Input Image	[512, 1024, 1]				
Conv-Layer 1	[512, 1024, 32]	Input Image	[3 × 3]	ReLU	True
Conv-Layer 2	[512, 1024, 32]	Conv-Layer 1	[3 × 3]	ReLU	True
Max-Pooling 1	[256, 512, 32]	Conv-Layer 2	[2 × 2]		
Conv-Layer 3	[256, 512, 64]	Max-Pooling 1	[3 × 3]	ReLU	True
Conv-Layer 4	[256, 512, 64]	Conv-Layer 3	[3 × 3]	ReLU	True
Max-Pooling 2	[128, 256, 64]	Conv-Layer 4	[2 × 2]		
Conv-Layer 5	[128, 256, 128]	Max-Pooling 2	[3 × 3]	ReLU	True
Conv-Layer 6	[128, 256, 128]	Conv-Layer 5	[3 × 3]	ReLU	True
Max-Pooling 3	[64, 128, 128]	Conv-Layer 6	[2 × 2]		
Conv-Layer 7	[64, 128, 256]	Max-Pooling 3	[3 × 3]	ReLU	True
Conv-Layer 8	[64, 128, 256]	Conv-Layer 7	[3 × 3]	ReLU	True
Max-Pooling 4	[32, 64, 256]	Conv-Layer 8	[2 × 2]		
Conv-Layer 9	[32, 64, 512]	Max-Pooling 4	[3 × 3]	ReLU	True
Conv-Layer 10	[32, 64, 512]	Conv-Layer 9	[3 × 3]	ReLU	True
Up-Sampling 1	[64, 128, 512]	Conv-Layer 10	[2 × 2]		
Conv-Layer 11	[64, 128, 256]	Up-Sampling 1	[3 × 3]	ReLU	True
Concatenate 1	[64, 128, 512]	Conv-Layer 8, Conv-Layer 11			
Conv-Layer 12	[64, 128, 256]	Concatenate 1	[3 × 3]	ReLU	True
Conv-Layer 13	[64, 128, 256]	Conv-Layer 12	[3 × 3]	ReLU	True
Up-Sampling 2	[128, 256, 256]	Conv-Layer 13	[2 × 2]		
Conv-Layer 14	[128, 256, 128]	Up-Sampling 2	[3 × 3]	ReLU	True
Concatenate 2	[128, 256, 256]	Conv-Layer 6, Conv-Layer 14			
Conv-Layer 15	[128, 256, 128]	Concatenate 2	[3 × 3]	ReLU	True
Conv-Layer 16	[128, 256, 128]	Conv-Layer 15	[3 × 3]	ReLU	True
Up-Sampling 3	[256, 512, 128]	Conv-Layer 16	[2 × 2]		
Conv-Layer 17	[256, 512, 64]	Up-Sampling 3	[3 × 3]	ReLU	True
Concatenate 3	[256, 512, 128]	Conv-Layer 4, Conv-Layer 17			
Conv-Layer 18	[256, 512, 64]	Concatenate 3	[3 × 3]	ReLU	True
Conv-Layer 19	[256, 512, 64]	Conv-Layer 18	[3 × 3]	ReLU	True
Up-Sampling 4	[512, 1024, 64]	Conv-Layer 19	[2 × 2]		
Conv-Layer 20	[512, 1024, 32]	Up-Sampling 4	[3 × 3]	ReLU	True
Concatenate 4	[512, 1024, 64]	Conv-Layer 2, Conv-Layer 20			
Conv-Layer 21	[512, 1024, 32]	Concatenate 4	[3 × 3]	ReLU	True
Conv-Layer 22	[512, 1024, 32]	Conv-Layer 21	[3 × 3]	ReLU	True
Conv-Layer 23	[512, 1024, 2]	Conv-Layer 22	[1 × 1]	Softmax	

Table 2. Network architecture of ROI and lesion segmentation model

Tooth-level classification model

Layer	Output Shape	Connected to	Kernel size	Activation	Batch Normalization
Input Image	[512, 1024, 1]				
Conv-Layer 1	[512, 1024, 32]	Input Image	[3 × 3]	ReLU	True
Conv-Layer 2	[512, 1024, 32]	Conv-Layer 1	[3 × 3]	ReLU	True
Max-Pooling 1	[256, 512, 32]	Conv-Layer 2	[2 × 2]		
Conv-Layer 3	[256, 512, 64]	Max-Pooling 1	[3 × 3]	ReLU	True
Conv-Layer 4	[256, 512, 64]	Conv-Layer 3	[3 × 3]	ReLU	True
Max-Pooling 2	[128, 256, 64]	Conv-Layer 4	[2 × 2]		
Conv-Layer 5	[128, 256, 128]	Max-Pooling 2	[3 × 3]	ReLU	True
Conv-Layer 6	[128, 256, 128]	Conv-Layer 5	[3 × 3]	ReLU	True
Max-Pooling 3	[64, 128, 128]	Conv-Layer 6	[2 × 2]		
Conv-Layer 7	[64, 128, 256]	Max-Pooling 3	[3 × 3]	ReLU	True
Conv-Layer 8	[64, 128, 256]	Conv-Layer 7	[3 × 3]	ReLU	True
Max-Pooling 4	[32, 64, 256]	Conv-Layer 8	[2 × 2]		
Conv-Layer 9	[32, 64, 512]	Max-Pooling 4	[3 × 3]	ReLU	True
Conv-Layer 10	[32, 64, 512]	Conv-Layer 9	[3 × 3]	ReLU	True
Conv-Layer 11	[32, 64, 1024]	Conv-Layer 10	[3 × 3]	ReLU	True
Conv-Layer 12	[32, 64, 1024]	Conv-Layer 11	[3 × 3]	ReLU	True
AveragePooling	[1024]	Conv-Layer 12	[32 × 64]		
Dropout 1 (0.8)	[1024]	AveragePooling			
Dense-Layer 1	[512]	Dropout 1		ReLU	
Dropout 2 (0.8)	[1024]	Dense-Layer 1			
Dense-Layer 2	[32]	Dropout 2		Sigmoid	

Table 3. Network architecture of tooth-level classification model.

Tooth-level performance comparison between the proposed method and dental clinicians

F1 score

**Upper Right**	11(I)	12(I)	13(C)	14(P)	15(P)	16(M)	17(M)	18(M)
No.	87/800	100/800	111/800	113/800	130/800	148/800	169/800	26/800
Clinician 1	0.613	0.652	0.664	0.642	0.678	0.801	0.803	0.807
Clinician 2	0.593	0.625	0.636	0.656	0.703	0.769	0.769	0.653
Clinician 3	0.587	0.624	0.629	0.650	0.701	0.759	0.793	0.627
Clinician 4	0.640	0.661	0.650	0.637	0.686	0.789	0.823	0.677
Clinician 5	0.653	0.629	0.679	0.651	0.664	0.730	0.793	0.754
Clinician Average	0.617	0.638	0.651	0.647	0.686	0.769	0.796	**0.703**
DeNTNet (Baseline)	0.615	0.660	0.689	0.690	0.612	0.699	0.668	0.185
DeNTNet	**0.757**	**0.734**	**0.765**	**0.780**	**0.761**	**0.820**	**0.829**	0.666
**Upper Left**	21(I)	22(I)	23(C)	24(P)	25(P)	26(M)	27(M)	28(M)
No.	83/800	90/800	95/800	97/800	125/800	147/800	147/800	18/800
Clinician 1	0.623	0.627	0.626	0.614	0.674	0.790	0.731	0.717
Clinician 2	0.572	0.577	0.616	0.611	0.659	0.748	0.700	0.731
Clinician 3	0.584	0.635	0.576	0.617	0.703	0.751	0.735	0.682
Clinician 4	0.635	0.628	0.580	0.577	0.673	0.797	0.770	0.560
Clinician 5	0.679	0.644	0.689	0.652	0.693	0.705	0.750	0.685
Clinician Average	0.618	0.622	0.617	0.614	0.680	0.758	0.737	**0.675**
DeNTNet (Baseline)	0.681	0.688	0.679	0.670	0.664	0.655	0.685	0.231
DeNTNet	**0.802**	**0.788**	**0.761**	**0.736**	**0.734**	**0.777**	**0.774**	0.545
**Lower Left**	31(I)	32(I)	33(C)	34(P)	35(P)	36(M)	37(M)	38(M)
No.	141/800	151/800	135/800	133/800	162/800	169/800	167/800	18/800
Clinician 1	0.646	0.649	0.612	0.623	0.700	0.775	0.772	0.651
Clinician 2	0.616	0.629	0.609	0.658	0.728	0.791	0.734	0.604
Clinician 3	0.628	0.657	0.591	0.607	0.713	0.774	0.822	0.666
Clinician 4	0.674	0.674	0.680	0.684	0.726	0.765	0.822	0.510
Clinician 5	0.670	0.659	0.624	0.658	0.706	0.814	0.840	0.705
Clinician Average	0.646	0.653	0.623	0.646	0.714	0.783	0.798	**0.627**
DeNTNet (Baseline)	0.662	0.681	0.703	**0.734**	0.719	0.713	0.641	0.162
DeNTNet	**0.772**	**0.770**	**0.722**	0.723	**0.787**	**0.818**	**0.791**	0.615
**Lower Right**	41(I)	42(I)	43(C)	44(P)	45(P)	46(M)	47(M)	48(M)
No.	142/800	145/800	127/800	130/800	151/800	156/800	140/800	22/800
Clinician 1	0.644	0.631	0.629	0.671	0.710	0.767	0.759	0.590
Clinician 2	0.619	0.644	0.622	0.655	0.726	0.781	0.760	0.608
Clinician 3	0.625	0.639	0.592	0.618	0.743	0.827	0.806	0.624
Clinician 4	0.690	0.676	0.616	0.672	0.727	0.834	0.786	0.754
Clinician 5	0.670	0.668	0.643	0.675	0.716	0.792	0.798	0.600
Clinician Average	0.649	0.651	0.620	0.658	0.724	0.800	0.781	0.635
DeNTNet (Baseline)	0.642	0.680	0.669	**0.760**	0.735	0.766	0.654	0.242
DeNTNet	**0.752**	**0.764**	**0.747**	0.744	**0.780**	**0.868**	**0.814**	**0.842**

Table 4. Comparison F1 score of proposed method to the dental clinicians on the test dataset. I:Incisor, C:Canine, P:Premolar, M:Molar.

Failure Cases

Failure cases in the third molar

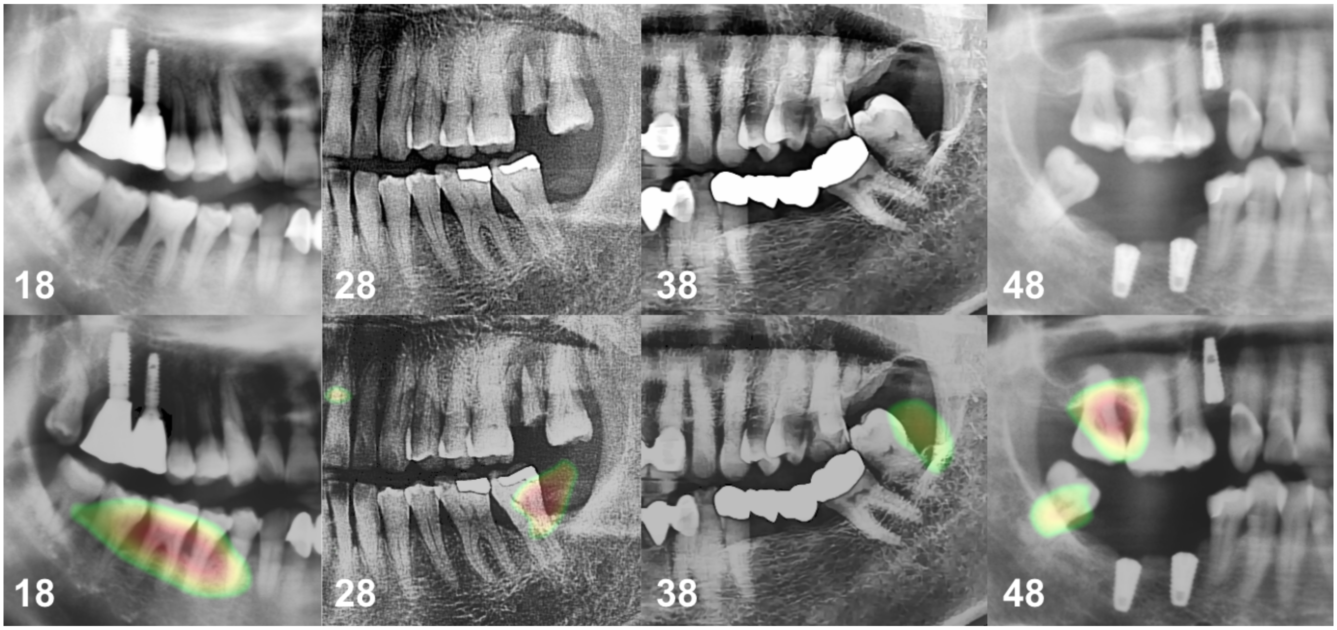

Figure 1. False negative cases of detecting PBL in the third molar. The output value of the model was lower than 0.1 for the third molar in these cases. **(18)** There was an implant next to the third molar with teeth number 18, **(28)** The third molar with teeth number 28 was next to a damaged tooth. **(38)** Rotated third molar with teeth number 38. **(48)** There was missing teeth next to the third molar with teeth number 48.
